# Supplementary material for: Maf-family bZIP transcription factor NRL interacts with RNA-binding proteins and R-loops in retinal photoreceptors
Source: eLife. 2025 Mar 6;13:RP103259. doi: 10.7554/eLife.103259 (PMC11884789; doi:10.7554/eLife.103259)
Supplement: Figure 1—figure supplement 1—source data 1. [file elife-103259-fig1-figsupp1-data1.zip › Figure 1 and Figure 1-figure supplement 1_Source Data 1/Figure 1 and Figure 1-figure supplement 1 _Source Data 1.pdf]

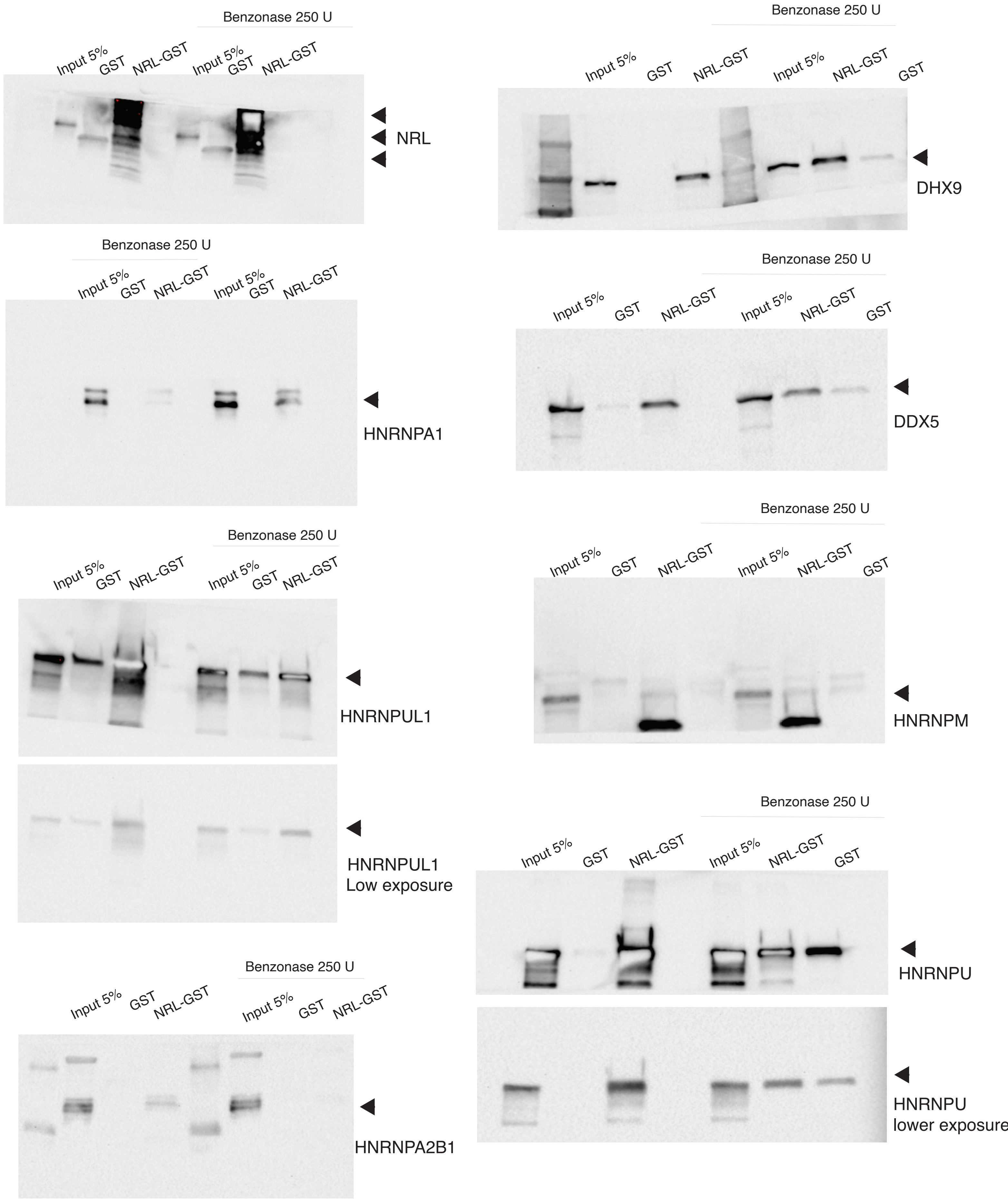

Figure 1 and Figure 1-supplement 1, Source Data 1. Original blots corresponding to Figure 1, Panel B and Figure 1-supplement 1, Panel A. Antibodies and Benzonase treatments are shown.
