# Supplementary figures and images for: Maf-family bZIP transcription factor NRL interacts with RNA-binding proteins and R-loops in retinal photoreceptors
Source: eLife. 2025 Mar 6;13:RP103259. doi: 10.7554/eLife.103259 (PMC11884789; doi:10.7554/eLife.103259)

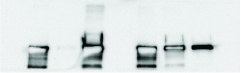

Supplement: Figure 1—figure supplement 1—source data 2. [file elife-103259-fig1-figsupp1-data2.zip › Figure 1 and Figure1-figure supplement 1_Source data 2/Figure 1 and Figure 1-figure supplement 1 Source Data 2_HNRNPU.jpg]

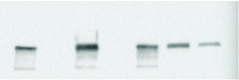

Supplement: Figure 1—figure supplement 1—source data 2. [file elife-103259-fig1-figsupp1-data2.zip › Figure 1 and Figure1-figure supplement 1_Source data 2/Figure 1 and Figure 1-figure supplement 1 Source Data 2_HNRNPU_low_exposure.jpg]

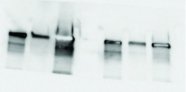

Supplement: Figure 1—figure supplement 1—source data 2. [file elife-103259-fig1-figsupp1-data2.zip › Figure 1 and Figure1-figure supplement 1_Source data 2/Figure 1 and Figure 1-figure supplement 1 Source Data 2_HNRNPUL1.jpg]

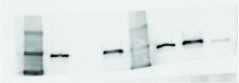

Supplement: Figure 1—figure supplement 1—source data 2. [file elife-103259-fig1-figsupp1-data2.zip › Figure 1 and Figure1-figure supplement 1_Source data 2/Figure 1 and Figure 1-figure supplement 1 Source Data 2_DHX9.jpg]

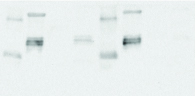

Supplement: Figure 1—figure supplement 1—source data 2. [file elife-103259-fig1-figsupp1-data2.zip › Figure 1 and Figure1-figure supplement 1_Source data 2/Figure 1 and Figure 1-figure supplement 1 Source Data 2_HNRNPA2B1.jpg]

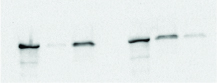

Supplement: Figure 1—figure supplement 1—source data 2. [file elife-103259-fig1-figsupp1-data2.zip › Figure 1 and Figure1-figure supplement 1_Source data 2/Figure 1 and Figure 1-figure supplement 1 Source Data 2_DDX5.jpg]

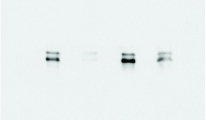

Supplement: Figure 1—figure supplement 1—source data 2. [file elife-103259-fig1-figsupp1-data2.zip › Figure 1 and Figure1-figure supplement 1_Source data 2/Figure 1 and Figure 1-figure supplement 1 Source Data 2_HNRNPA1.jpg]

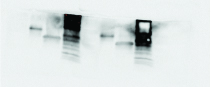

Supplement: Figure 1—figure supplement 1—source data 2. [file elife-103259-fig1-figsupp1-data2.zip › Figure 1 and Figure1-figure supplement 1_Source data 2/Figure 1 and Figure 1-figure supplement 1 Source Data 2_NRL.jpg]

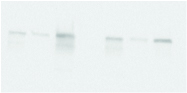

Supplement: Figure 1—figure supplement 1—source data 2. [file elife-103259-fig1-figsupp1-data2.zip › Figure 1 and Figure1-figure supplement 1_Source data 2/Figure 1 and Figure 1-figure supplement 1 Source Data 2_HNRNPUL1_low_exposure.jpg]

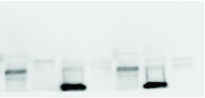

Supplement: Figure 1—figure supplement 1—source data 2. [file elife-103259-fig1-figsupp1-data2.zip › Figure 1 and Figure1-figure supplement 1_Source data 2/Figure 1 and Figure 1-figure supplement 1 Source Data 2_HNRNPM.jpg]

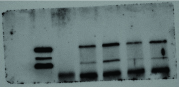

Supplement: Figure 4—source data 2. [file elife-103259-fig4-data2.zip › Figure4_Source data 2/Figure4A Source blot_DDX5.jpg]

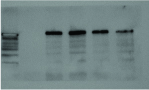

Supplement: Figure 4—source data 2. [file elife-103259-fig4-data2.zip › Figure4_Source data 2/Figure4A Source blot_DHX9.jpg]

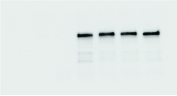

Supplement: Figure 4—source data 2. [file elife-103259-fig4-data2.zip › Figure4_Source data 2/Figure4E Source blot_DHX9.jpg]

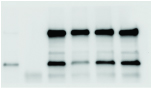

Supplement: Figure 4—source data 2. [file elife-103259-fig4-data2.zip › Figure4_Source data 2/Figure4E Source blot_DDX5.jpg]

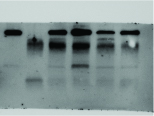

Supplement: Figure 4—source data 2. [file elife-103259-fig4-data2.zip › Figure4_Source data 2/Figure4B Source blot_DDX5jpg.jpg]

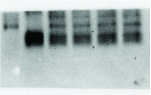

Supplement: Figure 4—source data 2. [file elife-103259-fig4-data2.zip › Figure4_Source data 2/Figure4E Source blot_NRL.jpg]

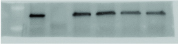

Supplement: Figure 4—source data 2. [file elife-103259-fig4-data2.zip › Figure4_Source data 2/Figure4F Source blot_DDX5.jpg]

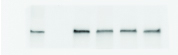

Supplement: Figure 4—source data 2. [file elife-103259-fig4-data2.zip › Figure4_Source data 2/Figure4F Source blot_DHX9.jpg]

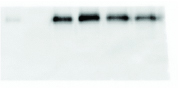

Supplement: Figure 4—source data 2. [file elife-103259-fig4-data2.zip › Figure4_Source data 2/Figure4F Source blot_NRL.jpg]

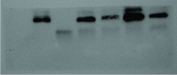

Supplement: Figure 4—source data 2. [file elife-103259-fig4-data2.zip › Figure4_Source data 2/Figure4A Source blot_NRL.jpg]

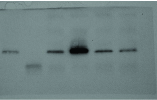

Supplement: Figure 4—source data 2. [file elife-103259-fig4-data2.zip › Figure4_Source data 2/Figure4B Source blot_NRL.jpg]

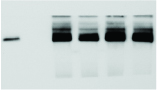

Supplement: Figure 4—source data 2. [file elife-103259-fig4-data2.zip › Figure4_Source data 2/Figure4B Source blot_DHX9.jpg]

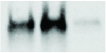

Supplement: Figure 5—source data 2. [file elife-103259-fig5-data2.zip › Figure 5A_source data 2/Figure 5A_source_data 2_NRL.jpg]

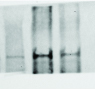

Supplement: Figure 5—source data 2. [file elife-103259-fig5-data2.zip › Figure 5A_source data 2/Figure5A_source data 2_DHX9.jpg]

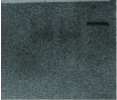

Supplement: Figure 5—source data 2. [file elife-103259-fig5-data2.zip › Figure 5A_source data 2/Figure5A_source data 2_DDX5.jpg]

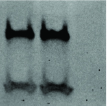

Supplement: Figure 5—source data 2. [file elife-103259-fig5-data2.zip › Figure 5A_source data 2/Figure 5A_source_data 2_IgG.jpg]

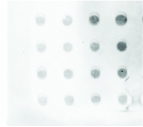

Supplement: Figure 6—source data 2. [file elife-103259-fig6-data2.zip › Figure 6A Source data 2/Figure 6A_source data 2_dsDNA.jpg]

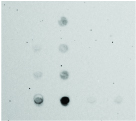

Supplement: Figure 6—source data 2. [file elife-103259-fig6-data2.zip › Figure 6A Source data 2/Figure 6A_source data 2_S9.6.jpg]

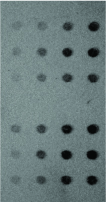

Supplement: Figure 6—figure supplement 1—source data 2. [file elife-103259-fig6-figsupp1-data2.zip › Figure 6-supplement 1_source data 2/Figure 6-supplement 1_source data 2_dsDNA.jpg]

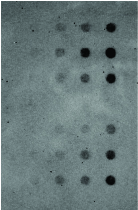

Supplement: Figure 6—figure supplement 1—source data 2. [file elife-103259-fig6-figsupp1-data2.zip › Figure 6-supplement 1_source data 2/Figure 6-supplement 1_source data 2_S9.6.jpg]
