# Supplementary material for: Maf-family bZIP transcription factor NRL interacts with RNA-binding proteins and R-loops in retinal photoreceptors
Source: eLife. 2025 Mar 6;13:RP103259. doi: 10.7554/eLife.103259 (PMC11884789; doi:10.7554/eLife.103259)
Supplement: Figure 4—source data 1. [file elife-103259-fig4-data1.zip › Figure4_source data 1/Figure4_source data 1.pdf]

Fig. 4A

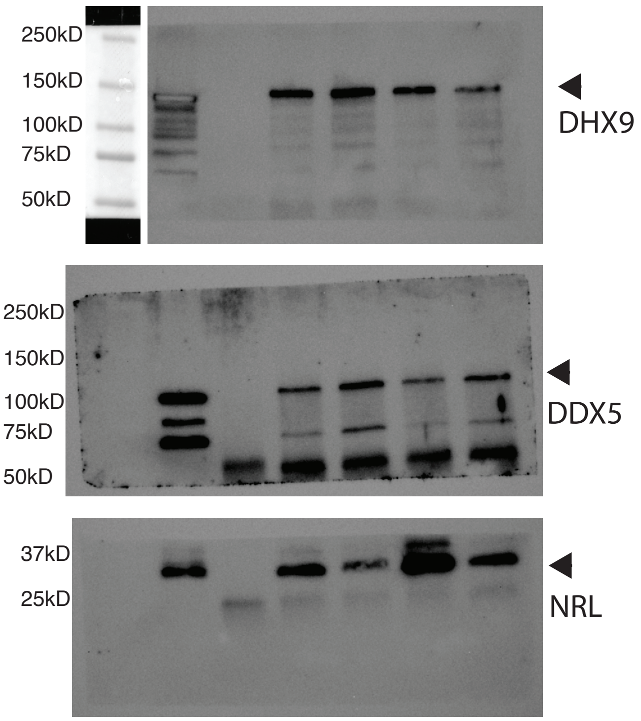

4B

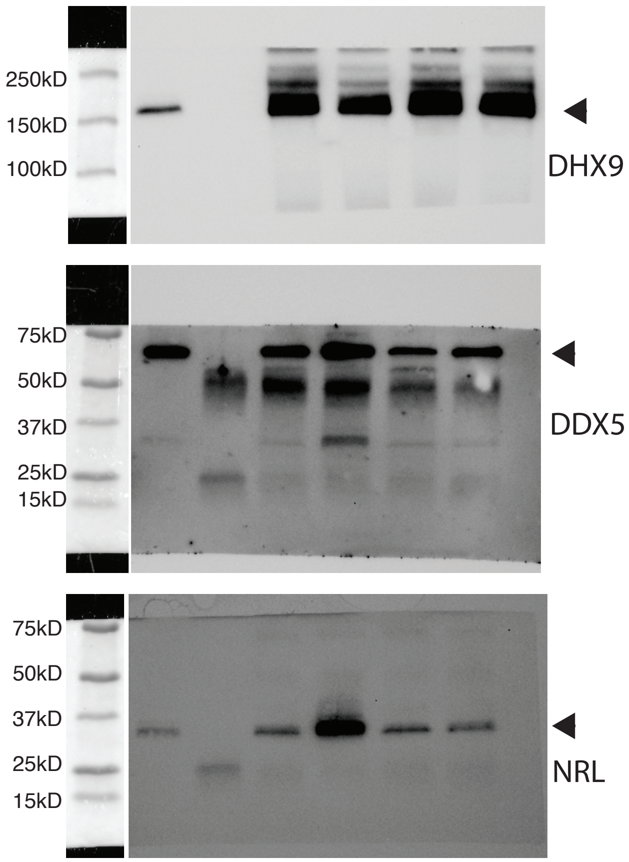

4E

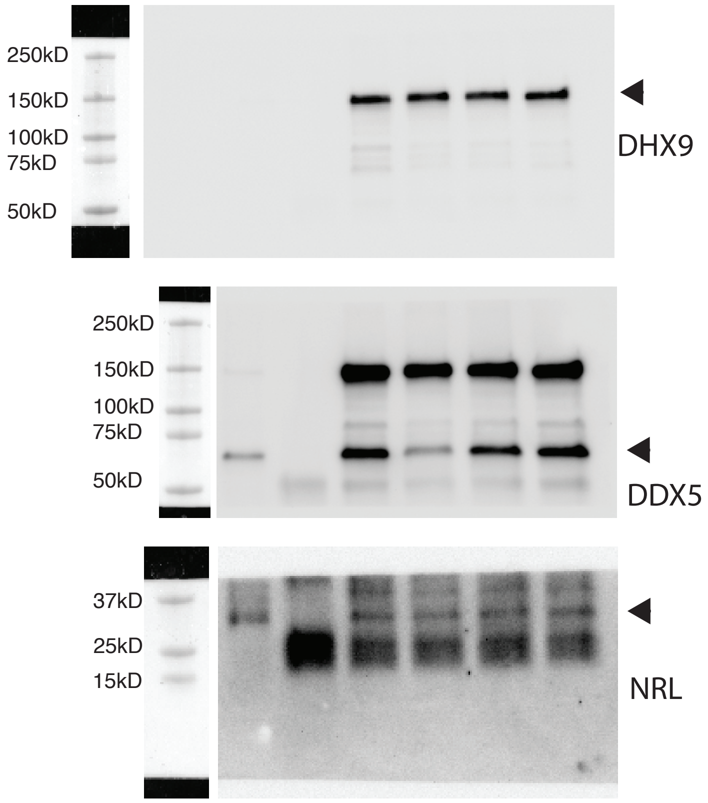

4F

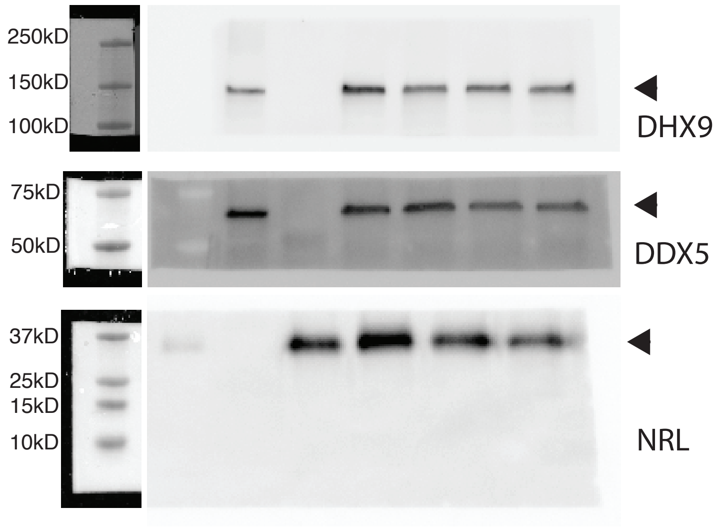

Figure 4, Source Data 1. Original blots corresponding to Figure 4, Panel A, B, E and F. Antibodies for inmunoblots are shown. Arrows indicate protein bands.
