# Supplementary material for: Maf-family bZIP transcription factor NRL interacts with RNA-binding proteins and R-loops in retinal photoreceptors
Source: eLife. 2025 Mar 6;13:RP103259. doi: 10.7554/eLife.103259 (PMC11884789; doi:10.7554/eLife.103259)
Supplement: Figure 5—source data 1. [file elife-103259-fig5-data1.zip › Figure 5A_source_data_1/Figure 5A_source_data 1.pdf]

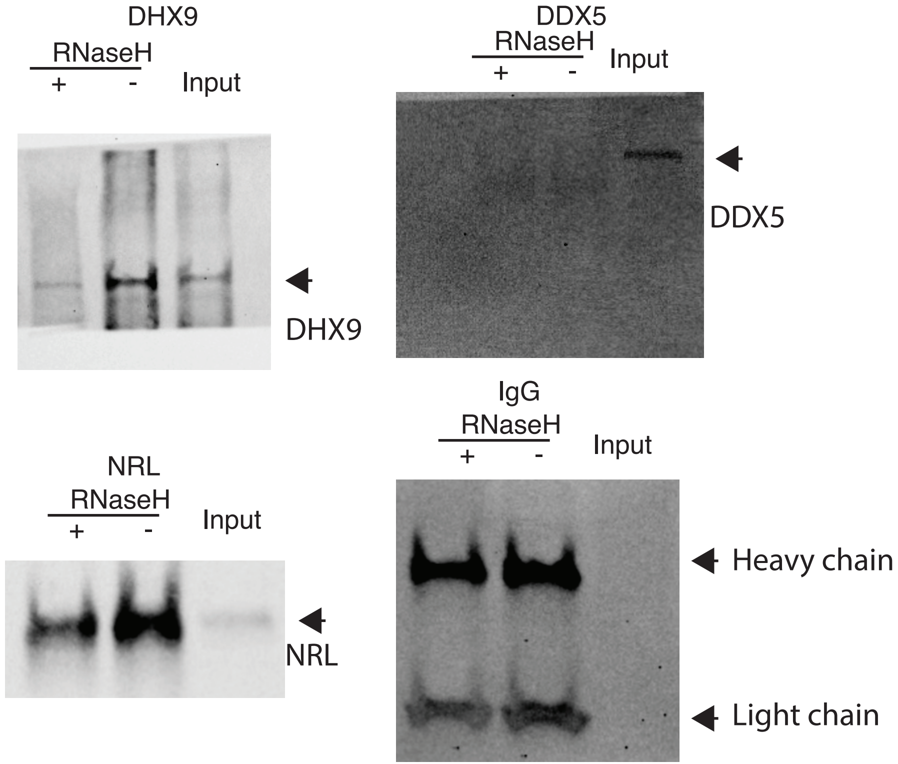

Figure 5, Source Data 1. Original blots corresponding to Figure 5, Panel A. Antibodies and RNaseH treatments are shown.
