# Supplementary material for: Maf-family bZIP transcription factor NRL interacts with RNA-binding proteins and R-loops in retinal photoreceptors
Source: eLife. 2025 Mar 6;13:RP103259. doi: 10.7554/eLife.103259 (PMC11884789; doi:10.7554/eLife.103259)
Supplement: Figure 6—source data 1. [file elife-103259-fig6-data1.zip › Figure 6A_source data 1/Figure 6A_source data 1.pdf]

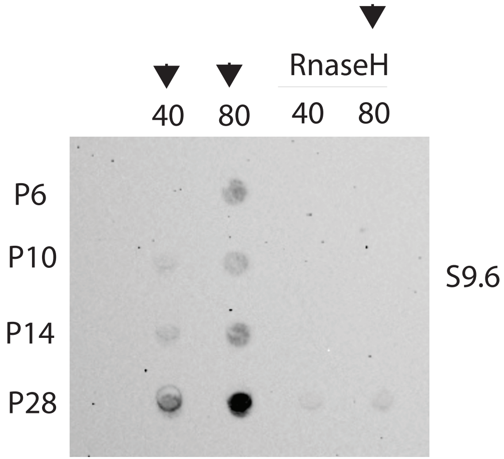

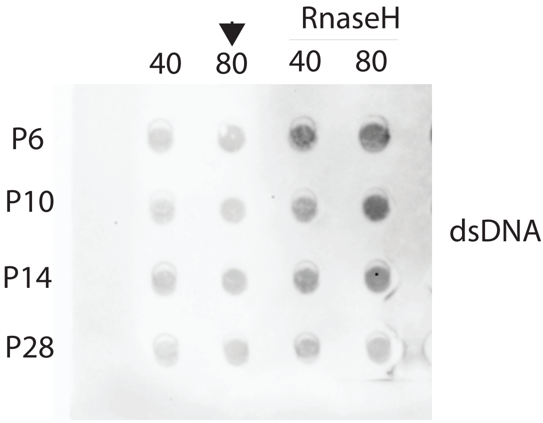

Figure 6, Source Data 1. Original blots corresponding to Figure 6, Panel A. Immunoblot using S9.6 and dsDNA antibodies are shown. RNaseH-treated samples are shown. Age is dysplayed as postnatal (P) days.
Arrows indicate lanes shown in Figure 6B.
