# Supplementary material for: Maf-family bZIP transcription factor NRL interacts with RNA-binding proteins and R-loops in retinal photoreceptors
Source: eLife. 2025 Mar 6;13:RP103259. doi: 10.7554/eLife.103259 (PMC11884789; doi:10.7554/eLife.103259)
Supplement: Figure 6—figure supplement 1—source data 1. [file elife-103259-fig6-figsupp1-data1.zip › Figure 6_supplement 1_source data 1/Figure 6-supplement 1_source_Data 1.pdf]

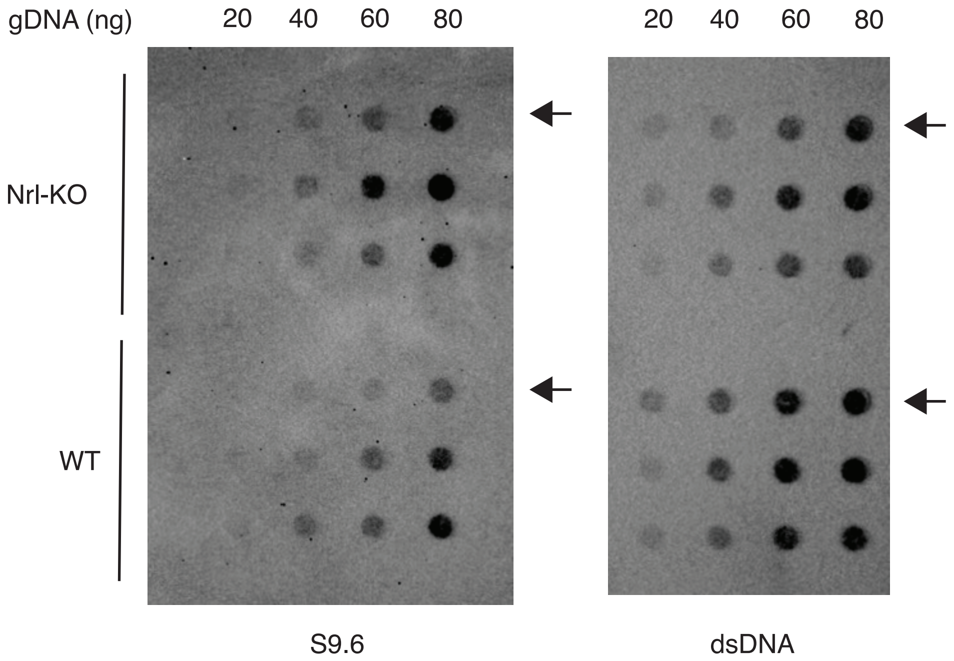

Figure 6-supplement 1, Source Data 1. Original blots corresponding to Figure 6-supplement 1. gDNA concentrations in nanograms are indicated on top panel. Rows indicate different samples. Arrows indicate samples displayed in image S5. Blots were incubated with S9.6 or dsDNA antibodies.
